# Synthesis and Characterization of New Chiral Smectic Four-Ring Esters

**DOI:** 10.3390/molecules29133134

**Published:** 2024-07-01

**Authors:** Magdalena Urbańska, Mateusz Gratzke, Michał Czerwiński

**Affiliations:** Institute of Chemistry, Military University of Technology, ul. Sylwestra Kaliskiego 2, 00-908 Warsaw, Poland; mateusz.gratzke@wat.edu.pl (M.G.); michal.czerwinski@wat.edu.pl (M.C.)

**Keywords:** synthesis, AFLCs, FLCs, multicomponent mixtures, helical pitch, electro-optical properties

## Abstract

Orthoconic antiferroelectric liquid crystals (OAFLCs) represent unique self-organized materials with significant potential for applications in photonic devices due to their sub-microsecond switching times and high optical contrast in electro-optical effects. However, almost all known OALFCs suffer from low chemical stability and short helical pitch values. This paper presents the synthesis and study results of two chiral AFLCs, featuring a four-ring structure in the rigid core and high chemical stability. The mesomorphic properties of these compounds were investigated using polarizing optical microscopy and differential scanning calorimetry. Spectrometry and electro-optical studies were employed to estimate the helical pitch, tilt angle, and spontaneous polarization of the synthesized compounds and the prepared mixtures. All studied compounds exhibit enantiotropic chiral smectic mesophases including the SmA*, the SmC*, and a very broad temperature range of the SmC_A_* phase. Doping top-modern antiferroelectric mixture with synthesized compounds offers benefits such as increased helical pitch and tilt angle values without significantly influencing spontaneous polarization. This allows the prepared mixture to be regarded as an OAFLC with high optical contrast, characterized by an almost perfect dark state. These valuable physicochemical and optical properties suggest significant potential of studied materials for practical applications.

## 1. Introduction

**Figure 1:**
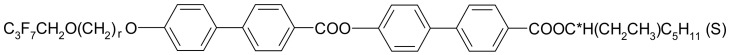
The general formula of the studied four-ring compounds (r = 3 and 7).

The ferroelectric (SmC*) and antiferroelectric (SmC_A_*) phases of liquid crystals have attracted significant attention from scientists due to their potential in modern technology [1,2,3,4]. Since the discovery of antiferroelectric liquid crystals [5], considerable effort has been devoted to designing new compounds and improving their electro-optical properties to prepare versatile materials for future applications. In addition to display applications, liquid crystals may be useful in laser beam steering, smart windows, and spatial modulators, among others [6,7,8,9,10,11,12,13,14,15,16]. Of particular note is the orthoconic antiferroelectric liquid crystals (OAFLCs), which consist of chiral molecules characterized by an angle of inclination of the molecules in the smectic layers equal or near-equal to 45° [17]. In this phase, the conic angle between the director of adjacent layers is nearly 90°. A surface-stabilized bookshelf structure of an OAFLC (SSOAFLC) is a negative optically uniaxial medium, with its extraordinary optical axis perpendicular to the boundary surfaces. Under zero electric field, a light beam impinging normally on the SSOAFLC slab (along the optical axis) propagates as in an isotropic medium. Typical structural defects within the surface stabilized structure of smectic phases, corresponding to local disorientation of the smectic layers, can degrade the optical contrast of such structures. However, in OAFLCs at normal incidence, the birefringence induced by this local disorientation cancels out, creating a perfect dark state and producing high optical contrast [18,19,20,21]. The OAFLCs hold promise for advanced electro-optical applications [22], and many scientists are actively searching for such materials to meet market demands. To optimize the physical parameters, such as temperature range, spontaneous polarization (Ps), helical pitch (p), and tilt angle (Θ) of AFLCs, adjustments are made to certain elements within the molecular structure. The most commonly used chiral center in antiferroelectrics is based on (S)-(+)-2-octanol [17,18,21,23,24,25]. Recently, several three-ring liquid crystals with a chiral center based on (S)-(+)-3-octanol have been synthesized, and their properties have been extensively characterized [26,27,28]. These compounds have the ferroelectric and/or antiferroelectric phases, good chemical stability, long helical pitch, and high tilt angles (some are orthoconic). However, their mixtures exhibit a maximum tilt angle of 43 degrees. Therefore, we synthesized and characterized two structurally similar rod-like compounds with an additional aromatic ring in the rigid core, as illustrated in Figure 1. These new compounds are designated as rPhPh, where r = 3 or 7.

This work aims to check how the additional (fourth) aromatic ring changes the mesomorphic, physicochemical, and electrooptical properties of AFLCs. To study the properties of new compounds, we applied various experimental techniques, such as differential scanning calorimetry (DSC), polarizing optical microscopy (POM), UV-Vis spectroscopy, tilt angle, and spontaneous polarization measurements. Finally, we investigated the influence of the synthesized compounds on the properties of a top-modern antiferroelectric mixture [29]. The results demonstrate that the compounds 3PhPh and 7PhPh exhibit a unique broad temperature range of the SmC_A_* phase and, most importantly, increase the tilt angle of the AFLC mixture, rendering it orthoconic with a higher helical pitch compared to before doping.

## 2. Results

### 2.1. Synthesis of Compounds

**Figure 2:**
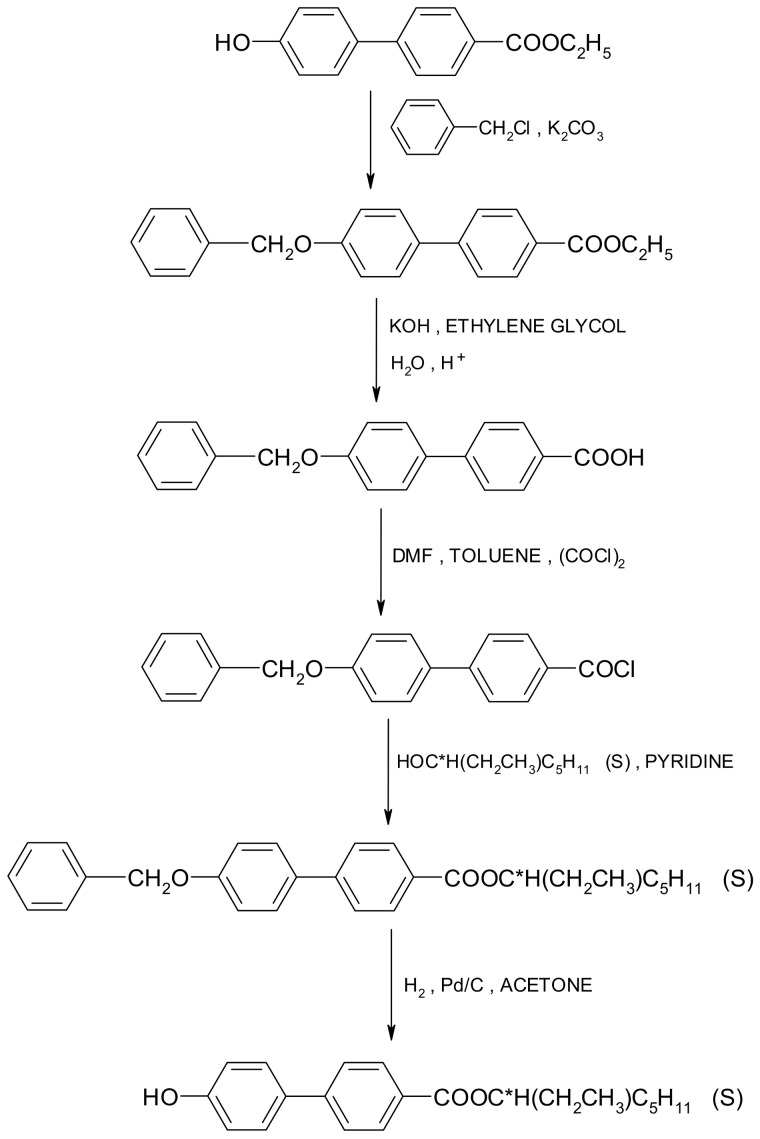
Synthetic route of chiral phenol.

**Figure 3:**
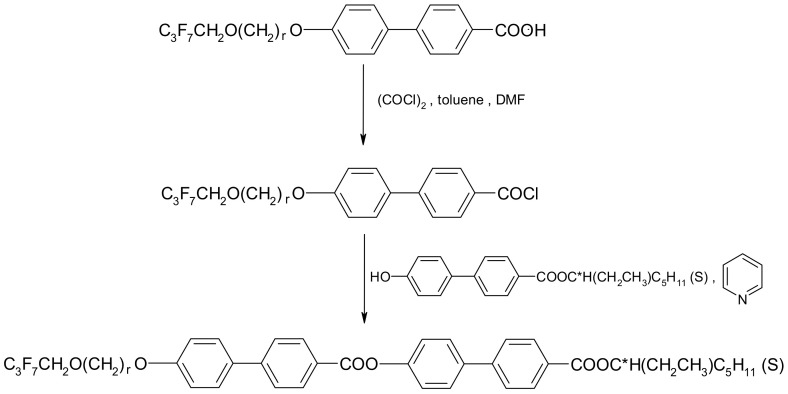
Synthetic route of four-ring chiral esters (r = 3 and 7).

The four-ring compounds were prepared using the same classical pathway by treating the chiral phenol (synthesized as shown in Figure 2) with a benzoic acid chloride in the presence of pyridine (see Figure 3). The synthesis is described in detail in Ref. [26]. The chiral liquid crystals were purified using a combination of column chromatography and recrystallization. The purity of the final compounds was checked using a Shimadzu prominence chromatograph with an SPD-M20A diode array detector. The compounds have a chemical purity higher than 99%. For ester 3PhPh MS: 786[M + Na]^+^ and 761[M − H]^−^ and for ester 7PhPh MS: 842[M + Na]^+^ and 817[M − H]^−^. The MS data for these esters are presented in Figures S1 and S2 in the Supplementary Materials. The yield of the esterification reaction was below 50% in all cases. The purity of the synthesized compounds was also monitored by thin-layer chromatography (TLC). The reaction course was described using the compound 3PhPh as an example.

STAGE I

In a three-necked round-bottom flask (100 mL) equipped with a thermocouple and a distillation head with a receptacle and a tube with calcium chloride and a magnetic stirrer was placed 1.135 g (2.5 mmol) 4-[3-(2,2,3,3,4,4,4-heptafluorobutoxy)prop-1-oxy]biphenylcarboxylic acid, 0.3 mL (2.75 mmol) of oxalyl chloride, and a drop of DMF and toluene (~50 mL). The mixture was then heated to ~30 °C and stirred at this temperature for approximately 4 h. The clear solution was heated to 111 °C to distill off excess oxalyl chloride along with toluene, and 4-[3-(2,2,3,3,4,4,4-heptafluorobutoxy)prop-1-oxy]biphenylcarboxylic acid chloride was formed.

STAGE II

After cooling the reaction, the distillation head was changed to a reflux condenser equipped with a tube with calcium chloride. Then, 0.815 g (2.5 mmol) of (S)-4-hydroxy-4′-(1-ethylhexyloxycarbonyl)biphenyl and 0.4 mL (5.0 mmol) of pyridine were added. Then, it was stirred for 16 h at ~60 °C. The mixture was cooled to room temperature and poured into 12 M hydrochloric acid (1 mL) and water (100 mL). The whole was filtered through a layer of activated carbon, the phases were separated, and then the organic phase was washed twice with water and dried over anhydrous magnesium sulfate. The drying agent was filtered off, and the solvent was distilled off on a vacuum evaporator. The product was dissolved in dichloromethane, purified by silica gel column chromatography, and crystallized from anhydrous ethanol. The second ester (r = 7) was obtained in the same way.

Elemental analysis of the synthesized compounds was conducted utilizing a Vario EL Cube apparatus (Elementar) working in CHNS mode. The elemental analysis (based on the traditional Pregl-Dumas method) allows rapid and accurate assessment of organic materials’ carbon, hydrogen, nitrogen, and sulfur content (wt.%). However, fluorinated chemicals are challenging to analyze via the CHNS analysis due to the high reactivity of fluorine. To prepare the compounds for analysis, samples of 2 mg ± 10% were wrapped in silver foil with ca. 2 mg of MgO powder. Silver and magnesium oxide strongly bind fluorine, and hence, they suppress the formation of interferents (such as SiF_4_ and CO_2_F_2_), as well as highly reactive fluorine species (such as HF), that potentially could damage elements of the apparatus made from quartz. Each sample was analyzed twice, and the result was calculated as an average. For nitrogen- and sulfur-free compounds, the sum of fluorine and oxygen content can be estimated as O% + F% = 100% − C% − H% (assuming that the analyzed material does not contain other impurities). The elemental analysis results are provided in Table 1: the elemental analysis calculation for the compound 3PhPh − **C_41_H_41_F_7_O_6_**: C, 64.56; H, 5.42; F, 17.44; O, 12.59 and for the compound 7PhPh − **C_45_H_49_F_7_O_6_**: C, 66.00; H, 6.03; F, 16.24; O, 11.72.

Proton (^1^H) and carbon (^13^C) nuclear magnetic resonance (NMR) spectra in CDCl_3_ were collected using a Bruker Avance III 500 MHz spectrometer. Comparing the NMR spectra confirmed the compliance of real structures with the planned structures (see Figures S3–S6 in the Supplementary Materials). The values of chemical shifts for the synthesized esters are given in Table 2 and Table 3. Protons are marked as shown below:


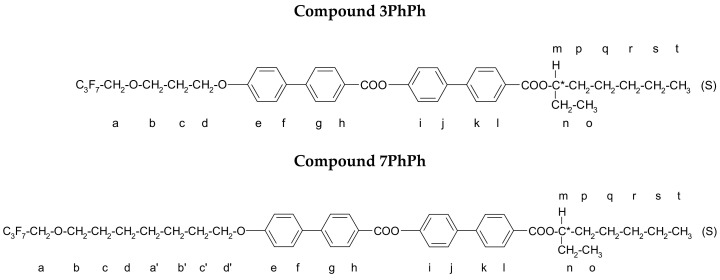


**Table 2:** ^1^H NMR chemical shift data (in ppm) for the esters in CDCl_3_ solution.

Protons	Compound 3PhPh	Compound 7PhPh
**a**	3.965 (2H, t)	3.944 (2H, t)
**b**	3.841 (2H, t)	3.632 (2H, t)
**c**	2.152 (2H, m)	1.529–1.877 (10H, m)
**d**	4.145 (2H, t)	
**a’**		
**b’**		
**c’**		
**d’**		3.972 (2H, t)
**e**	7.033 (2H, d)	7.026 (2H, d)
**f**	8.245 (2H, d)	8.176 (2H, d)
**g**	7.362 (2H, d)	7.359 (2H, d)
**h**	7.632 (2H, d)	7.625 (2H, d)
**i**	7.707 (2H, d)	7.691(2H, d)
**j**	7.724 (2H, d)	7.722 (2H, d)
**k**	8.161 (2H, d)	8.159 (2H, d)
**l**	8.301 (2H, d)	8.296 (2H, d)
**m**	5.142 (1H, m)	5.140 (1H, m)
**n**	1.351 (2H, t)	1.341 (2H, t)
**o**	1.423 (3H, d)	1.348 (3H, d)
**p**	1.337–1.780 (8H, m)	1.336–1.434 (8H, m)
**q**		
**r**		
**s**		
**t**	0.929 (3H, t)	0.987 (3H, t)

**Table 3:** Values of chemical shifts for the chiral center (atoms “m”) of the esters in ^1^H and ^13^C NMR spectra.

**Compound 3PhPh**	5.142 67.770	^1^H NMR [ppm] ^13^C NMR [ppm]
**Compound 7PhPh**	5.140 67.773	^1^H NMR [ppm] ^13^C NMR [ppm]

### 2.2. Mesogenic Properties of Compounds

Transition temperatures and enthalpies of the transition for the compounds 3PhPh and 7PhPh were determined by differential scanning calorimetry (DSC) using a SETARAM 141 microcalorimeter at the heating/cooling rate of 2 °C·min^−1^. Mesophases were identified by observing the textures using an OLYMPUS BX51 optical microscope under crossed polarizers with a Linkam THMS-600 hot stage controlled by a TMS-93 temperature programmer. All the phases were easy to recognize from the microscopic textures. Phase transition temperatures of the synthesized compounds are presented in Table 4.

The SmC_A_*-SmC* transition exhibits small enthalpy values below 0.07 kJ·mol^−1^, whereas the transition from smectic C* to smectic A* shows higher enthalpies exceeding 1 kJ·mol^−1^. Both compounds exhibit relatively low melting enthalpy, which could be advantageous for their solubility with other liquid crystals in the preparation of the mixtures.

**Figure 4:**
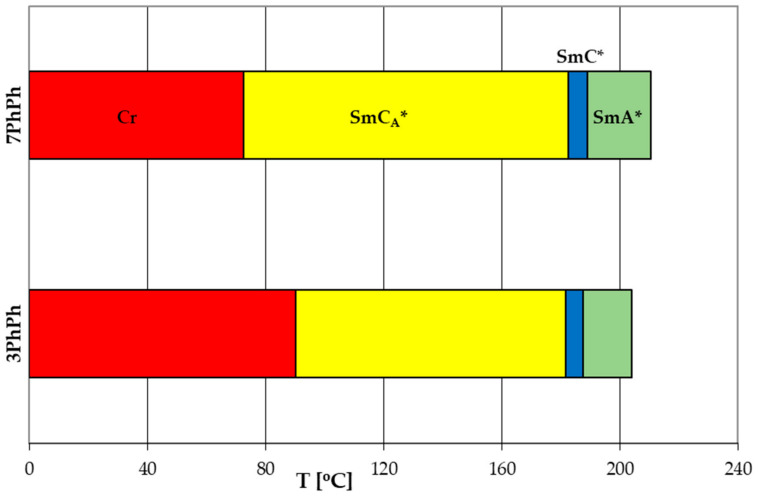
Comparison of phase transition temperatures for the compounds 3PhPh and 7PhPh.

**Figure 5:**
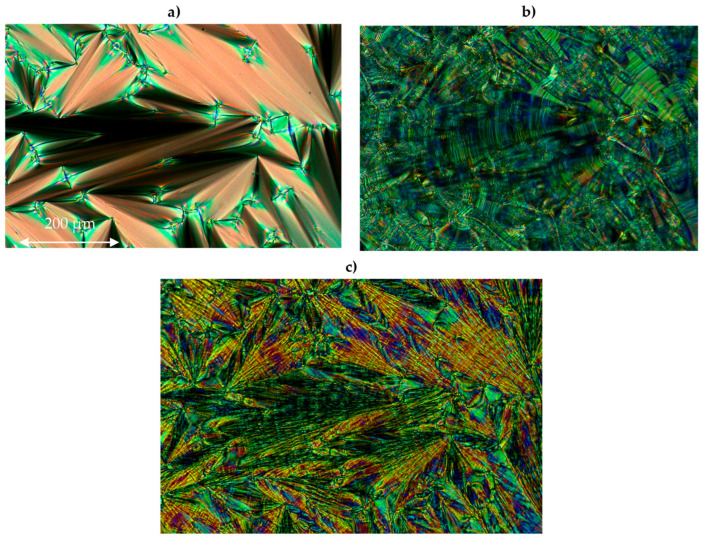
The photos of the microscopic pattern for the compound 3PhPh obtained during the cooling cycle: (**a**) in the SmA* phase at 203.3 °C, (**b**) in the SmC* phase at 189.8 °C, and (**c**) in the SmC_A_* phase at 43.7 °C; a width of all the microphotographs is about 600 μm.

The observed phase sequence is as follows: Cr-SmC_A_*-SmC*-SmA*-Iso, see Figure 4. The compounds 3PhPh and 7PhPh exhibit a very broad temperature range of the SmC_A_* phase. The SmC* phase is observed within a very narrow temperature range, while the SmA* phase appears in a medium temperature range. The compound 3PhPh has the lowest melting point among them. Both compounds exhibit high clearing points (above 200 °C). Characteristic microscopic patterns corresponding to the observed phases are shown in Figure 5a–c.

**Figure 6:**
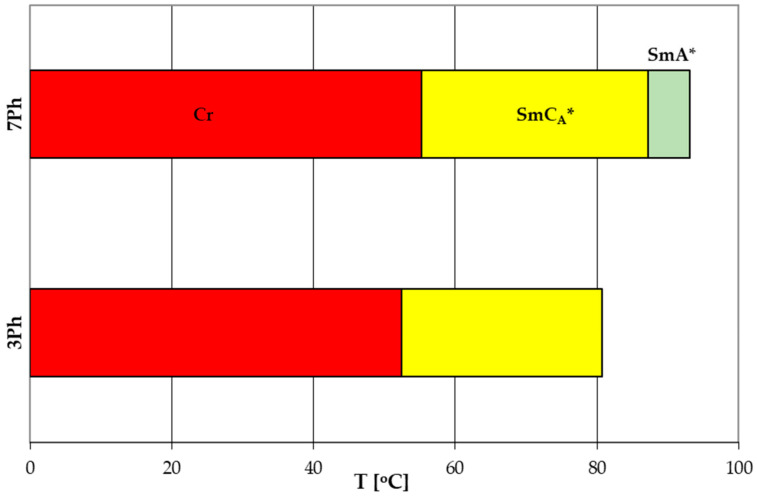
Comparison of phase transition temperatures for the chiral analogs of the compounds 3PhPh and 7PhPh but with three aromatic rings.

**Figure 7:**
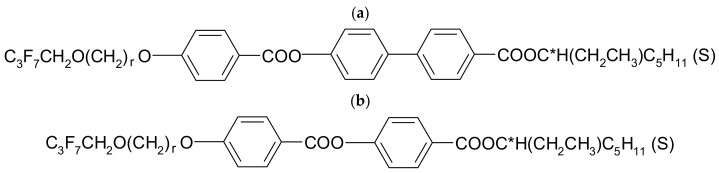
The general formulas of the two- (**b**) and three-ring (**a**) compounds (r = 3 and 7).

For comparison, Figure 6 [26,27] shows phase transition temperatures for analogs of the compounds 3PhPh and 7PhPh but with three aromatic rings (the acronym used to designate this type of compound in this work is rPh). Their general formula is presented in Figure 7a.

Compounds with three aromatic rings show significantly lower clearing points (below 100 °C) and a two times shorter range of the antiferroelectric phase compared to studied four-ring compounds. The SmA* phase appears for the compound with a longer oligomethylene spacer. No ferroelectric phase was observed for any three-ring compound. Additionally, two-ring compounds were synthesized (Figure 7b); however, they did not exhibit liquid crystalline properties, highlighting the significant influence of the number of aromatic rings on the occurrence of mesomorphic phases.

### 2.3. Electro-Optical and Physicochemical Properties of Mixtures

The electro-optical method was used to measure the tilt angle of the director [30,31]. The measurements were made using the PZO 02198 polarizing microscope with a Linkam HFS 91 heating stage and a Linkam TMS 93 temperature controller, a digital oscilloscope (R&S HM0724), and a photodetector (THORLABS PDA100A-EC) with a silicon diode.

The triangular wave method was used to measure the spontaneous polarization of studied materials [32] in a setup similar to that for tilt angle measurements. The used cells for the above electro-optical measurements were 1.6 μm thick with homogeneous alignment. This surface was achieved by spin-coating Nylon 6.6 on the indium tin oxide-coated glass, followed by curing and rubbing steps. All measurements were conducted upon cooling. The measurements were carried out in the temperature range from 98 °C to 30 °C, decreasing the temperature by 2 °C to 70 °C and then by 5 °C.

Due to the phase transition to the isotropic phase occurring at temperatures higher than 200 °C, filling the electro-optical cell with the studied compounds was impossible. Therefore, the electro-optical properties were measured for two mixtures prepared by adding 20 wt% of either compound 3PhPh or 7PhPh to the eutectic mixture W-450 (base mixture), as shown in Table 5 and Table 6. The composition of the eutectic mixture was calculated from equations given by Le Chatelier, Schröder, and van Laar. These equations combine component concentrations in the individual phases from temperature and parameters of the pure compounds (temperature and enthalpy of the phase transformations), assuming that the system is in the isobaric conditions, as well as constant of the thermal capacity of components in the phases being in the phase equilibrium, and lack of chemical reaction of components in the system [33,34,35,36]. Furthermore, the design of new single-molecule materials does not always result in the desired mesomorphic and electro-optic properties. Therefore, preparing multicomponent mixtures is a highly effective strategy, particularly for achieving FLC and AFLC behaviors [37,38,39,40,41,42,43,44,45,46,47].

**Figure 8:**
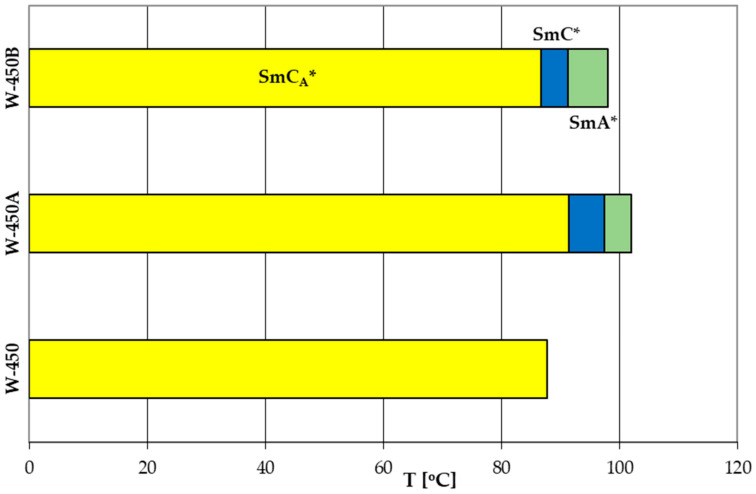
Comparison of phase transition temperatures for the mixtures W-450, W-450A, and W-450B.

A comparison of phase transition temperatures for the base mixture and its modification with the compounds 3PhPh and 7PhPh is shown in Figure 8.

The addition of the compound 3PhPh or the compound 7PhPh extends the temperature range of the antiferroelectric phase in the new mixtures. The mixtures W-450A and W-450B also exhibit a short range of the SmC* and the SmA* phases. All mixtures have very low melting points (below 0 °C) and clearing points below 105 °C. To confirm the antiferroelectric behavior of the studied mixtures, the quasistatic electro-optical characteristics were recorded with triangular driving voltage pulses at a frequency of f = 0.1 Hz. Each mixture exhibits typical for the SmC_A_* phase double hysteresis (see example characteristics in Figure S7 in the Supplementary Materials).

**Figure 9:**
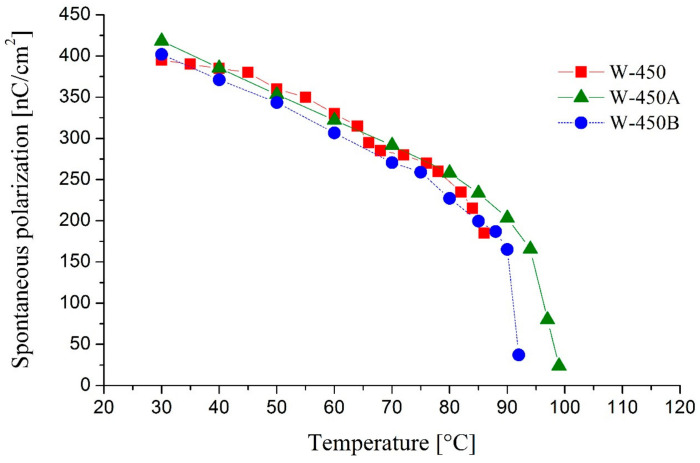
The temperature dependence of the spontaneous polarization for the mixtures W-450, W-450A, and W-450B.

**Figure 10:**
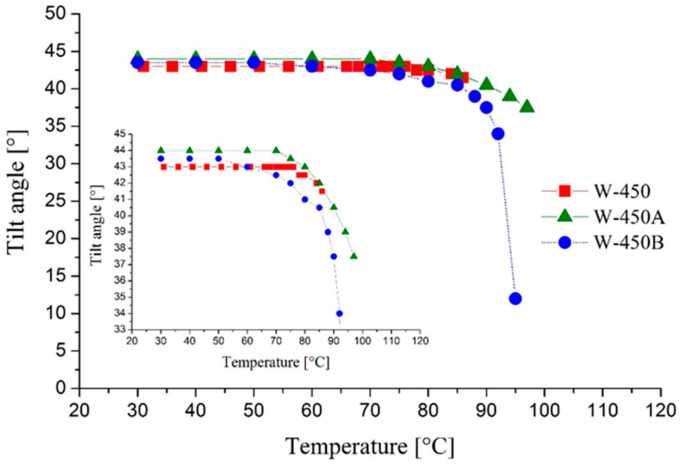
The temperature dependence of the director’s tilt angle for the mixtures W-450, W-450A, and W-450B.

Figure 9 and Figure 10 compare the temperature dependencies of the director’s spontaneous polarization and tilt angle for the mixtures W-450A and W-450B with the base mixture W-450, respectively.

The temperature dependence of spontaneous polarization (P_S_) for all studied mixtures is typical for AFLCs, showing an increase in P_S_ upon cooling. The spontaneous polarization values of the mixtures do not differ significantly and range from 390 to 420 nC/cm^2^ at 30 °C.

**Figure 11:**
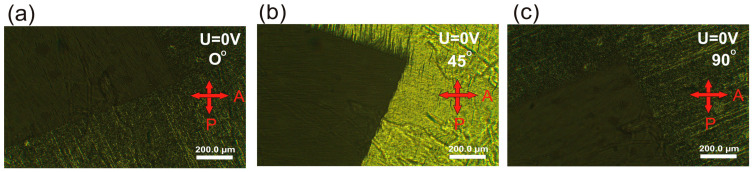
Microphotographs of the electro-optical cell filled with the mixture W-450A between crossed polarizers in the dark state without electric field in surface-stabilized geometry for the angle between polarizer and layer normal: (**a**) 0°, (**b**) 45°, and (**c**) 90°.

The mixtures doped with the compounds 3PhPh and 7PhPh exhibit slightly higher tilt angles compared to the base mixture W-450. The maximum tilt angle observed is 44° for the mixture W-450A, while the mixture W-450B has a tilt angle that is 0.5 degrees lower. It is important to note that even a slight increase in the tilt angle above 43 degrees leads to a significant rise in contrast for SSAFLCs [17]. In this context, the mixture W-450A can be considered nearly orthoconic or orthoconic. To confirm this behavior, we captured POM images of the electro-optic cell filled with the mixture W-450A and aligned in a bookshelf geometry achieved through surface stabilization (Figure 11). Rotating the cell between crossed polarizers shows no change in the dark state of the cell’s active surface. The same experiment with the mixture W-450B gives similar results. This behavior is characteristic of an optically isotropic material, such as OAFLCs [19,20,21].

**Figure 12:**
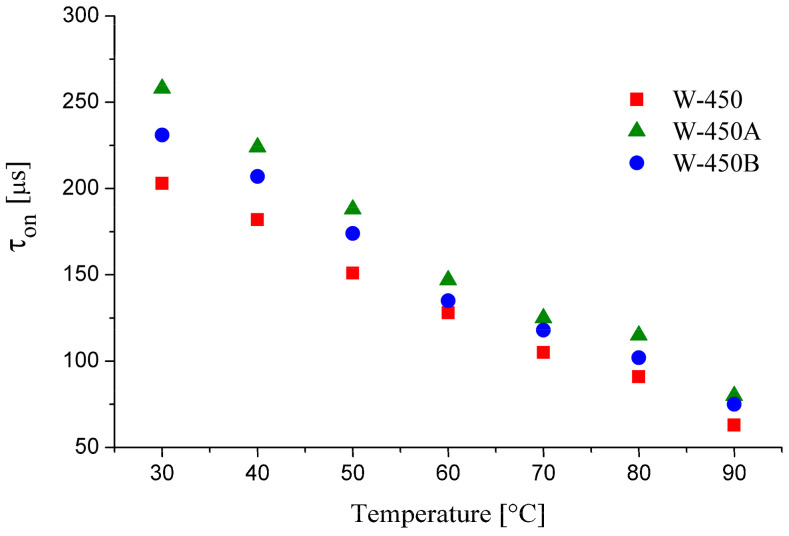
The switching ON time (τ_on_) as a function of temperature for the studied mixtures.

The temperature dependence of the switching ON time (τ_on_) was measured by using a special driving waveform I [48,49] for evaluation of dynamic electro-optical characteristics and is presented in Figure 12. A slight increase in τ_on_ is observed for the doped mixtures compared to the base mixture W-450 across the entire temperature range of the SmC_A_* phase. This is most likely due to the higher viscosity of the mixtures containing compounds with four benzene rings in the rigid core. However, the switching time for each mixture remains below 300 µs, which is a significant advantage over any known nematic liquid crystals.

### 2.4. Helical Pitch of Compounds and Mixtures

The helical pitch measurements were performed based on the selective light reflection phenomenon [42,50,51]. Before measurements, a thin layer of orienting surfactant was applied on the glass plate to force the liquid crystal molecules’ required homeotropic alignment (perpendicular to the surface). Such slides were used to register a baseline on a UV-Vis-NIR spectrophotometer (SHIMADZU 3600) in the 360–3000 nm wavelength range. After baseline collection, the studied materials were applied on the surface of the slide and the spectra were recorded. The measurements were performed in a cooling cycle. The spectrophotometer was equipped with a U7 MLW temperature controller. The pitch p was calculated for the antiferroelectric phase from the dependence,

λ_max_ = *n*·p (1)

and for the ferroelectric phase from the dependence,

λ_max_ = 2*n*·p (2)

where *n* is the average refractive index. The value *n* = 1.5 was taken into calculation [52].

It was impossible to measure the helical pitch for compound 7PhPh because the results were above the measuring range of the used spectrophotometer; the same situation was observed for the compound with three aromatic rings—7Ph [27].

**Figure 13:**
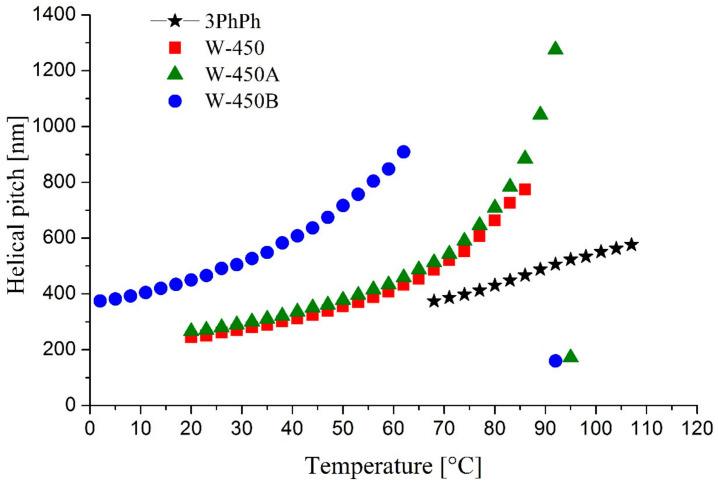
The helical pitch dependence on temperature for the compound 3PhPh and the mixtures W-450A, W-450A, and W-450B.

The results of the helical pitch measurements for compound 3PhPh and all mixtures are presented in Figure 13.

In the ferroelectric phase, the helical pitch observed for the mixtures W-450A and W-450B is very short (below 200 nm). In the antiferroelectric phase, the helical pitch increases with temperature. The highest observed value of the helical pitch for compound 3PhPh is 576 nm at 107 °C. The compound 3Ph has a slightly shorter helical pitch in the antiferroelectric phase (the maximum value is 520 nm at 74 °C) [26]. A longer pitch is observed for the doped mixtures than for the base mixture. The longest pitch is observed for the mixture W-450A (above 1300 nm) at higher temperatures. On the other hand, the mixture W-450B exhibits the highest values of the helical pitch at lower temperatures. The mixture W-450A has helical pitch values in the SmC_A_* phase similar to those of the base mixture W-450. When AFLC is used, it is important to control the dependence of the helical structure on temperature because, in the SmC_A_* phase, the helical structure of the molecules depends strongly on temperature, as shown in Figure 13, unlike the SmC* phase, where this dependence is usually independent of temperature [53].

## 3. Conclusions

In this work, we synthesized and studied chiral liquid crystalline esters containing four aromatic rings. We modified previously studied structures [26,27] and introduced an additional aromatic ring. These compounds show a broad temperature range of the antiferroelectric phase, two times wider than three-ring analogs. The synthesized materials also exhibit the paraelectric orthogonal and tilted ferroelectric phases.

Additionally, synthesized compounds were used as dopants for the top-modern high-tilted antiferroelectric mixture W-450. The prepared mixtures W-450A and W-450B show the antiferroelectric (SmC_A_*) phase in a broader temperature range and higher tilt angle (above 43° at low temperatures) compared to the base mixture W-450. The values of spontaneous polarization are similar for all studied mixtures. The mixtures’ helical pitch in the SmC_A_* phase is relatively long. Two doped mixtures have a longer pitch than the base mixture W-450. Moreover, we have proven the orthoconic nature of the prepared mixtures with the synthesized compounds.

The obtained results show the strong influence of the number of aromatic rings on the compounds’ mesomorphic and electro-optical properties.

As previously proven, liquid crystalline materials with four aromatic rings generally exhibit very high phase transition temperatures [38,54,55,56,57,58,59,60,61], making most applications impossible. Nevertheless, these compounds work great as components or dopants in multicomponent mixtures. The synthesized compounds are suitable as dopants for AFLC mixtures, enhancing their performance properties, thereby making them suitable materials for constructing electro-optic transducers with fast response times and high optical contrast, even though these are structures with four aromatic rings and high clearing points.

## Figures and Tables

**Table 1:** The elemental analysis of the synthesized compounds.

Chemical Formula (and Acronym) of the Compound	C [%]	H [%]	O + F [%]
**C_41_H_41_F_7_O_6_ (3PhPh)**	64.21	4.935	30.855
**C_45_H_49_F_7_O_6_ (7PhPh)**	67.48	5.948	26.572

**Table 4:** Phase transition temperatures [°C] and enthalpies (italics) for the compounds 3PhPh and 7PhPh.

Acronym	Cr		SmC_A_*		SmC*		SmA*		Iso
		-		176.0–177.7		195.3–196.6		209.8–211.7	
		90.2		181.7		187.7		204.2	
**3PhPh**	•	41.8	•	177.3	•	186.1	•	205.5	•
		*16.82*		*0.06*		*1.15*		*1.82*	
		-		186.7–187.5		194.0–195.5		215.0–217.8	
		72.7		182.6		189.1		210.4	
**7PhPh**	•	-	•	179.6	•	188.9	•	209.8	•
		*14.78*		*0.04*		*1.04*		*2.13*	

The first row is temperatures from microscopic observations obtained in the heating cycle; the second row is temperatures from differential scanning calorimetry obtained in the heating cycle; the third row is temperatures from differential scanning calorimetry obtained in the cooling cycle; the fourth row is enthalpy, ΔH in the kJ·mol^−1^ obtained in the heating cycle.

**Table 5:** The composition of the base mixture W-450.

No.	Compounds	Concentration [wt%]
**1.**	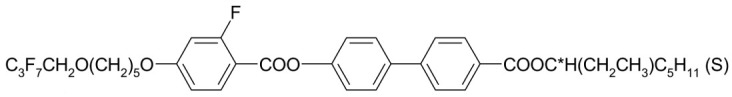 [26]	13.0
**2.**	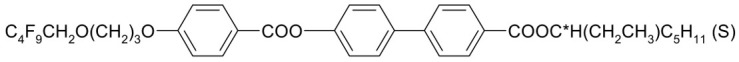 [26]	45.0
**3.**	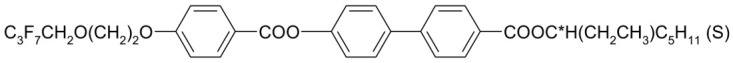 [28]	42.0

**Table 6:** The compositions of the prepared mixtures.

Acronym of the Mixture	Mixture W-450 Concentration [wt%]	Dopant and Concentration [wt%]
**W-450A**	80.0	**3PhPh**; 20.0
**W-450B**	80.0	**7PhPh**; 20.0

## Data Availability

The data presented in this study are available on request from the corresponding author.

## Supplementary Materials

The following supporting information can be downloaded at: https://www.mdpi.com/article/10.3390/molecules29133134/s1, Figure S1. Mass spectrum of the compound 3PhPh; Figure S2. Mass spectrum of the compound 7PhPh; Figure S3: ^1^H NMR spectrum of the compound 3PhPh in CDCl_3_; Figure S4: ^13^C NMR spectrum of the compound 3PhPh in CDCl_3_; Figure S5: ^1^H NMR spectrum of the compound 7PhPh in CDCl_3_; Figure S6: ^13^C NMR spectrum of the compound 7PhPh in CDCl_3_; Figure S7. Quasistatic electro-optical response for the mixture W-450A, under application of a triangular waveform at 0.1 Hz.

## References

[1] Vertogen G., de Jeu W.H. (1988). Thermotropic Liquid Crystals, Fundamentals.

[2] Lagerwall S.T. (1999). Ferroelectric and Antiferroelectric Liquid Crystals.

[3] Meyer R.B. (1977). Ferroelectric liquid crystals; a review. Mol. Cryst. Liq. Cryst..

[4] Fukuda A., Takanishi Y., Isozaki T., Ishikawa K., Takezoe H. (1994). Antiferroelectric chiral smectic liquid crystals. J. Mat. Chem..

[5] Chandani A.D.L., Górecka E., Ouchi Y., Takezoe H., Fukuda A. (1989). Antiferroelectric chiral smectic phases responsible for the tristable switching in MHPOBC. Jpn. J. Appl. Phys..

[6] Clark N.A., Lagerwall S.T. (1980). Submicrosecond bistable electro-optic switching in liquid crystals. Appl. Phys. Lett..

[7] D’have K., Rudquist P., Lagerwall S.T., Pauwels H., Drzewiński W., Dąbrowski R. (2000). Solution of the dark state problem in antiferroelectric liquid crystal displays. Appl. Phys. Lett..

[8] Otón J.M., Quintana X., Castillo P.L., Lara A., Urruchi V., Bennis N. (2004). Antiferroelectric liquid crystal displays. Opto-Electr. Rev..

[9] Lagerwall J.P.F., Scalia G. (2012). A new era for liquid crystal research: Applications of liquid crystals in soft matter nano-, bio- and microtechnology. Curr. Appl. Phys..

[10] Zhang Y.-S., Liu C.-Y., Emelyanenko A.V., Liu J.-H. (2018). Synthesis of predesigned ferroelectric liquid crystals and their applications in field-sequential color displays. Adv. Funct. Mater..

[11] Guo Q., Xu L., Sun J., Yang X., Liu H., Yan K., Zhao H., Chigrinov V.G., Kwok H.S. (2019). Fast switching beam steering based on ferroelectric liquid crystal phase shutter and polarisation grating. Liq. Cryst..

[12] Jiang B., Liu L., Gao Z., Feng Z., Zheng Y., Wang W. (2019). Fast dual-stimuli-responsive dynamic surface wrinkles with high bistability for smart windows and rewritable optical displays. ACS Appl. Mater. Interfaces.

[13] Fuh A.Y.G., Chih S.Y., Wu S.T. (2018). Advanced electro-optical smart window based on PSLC using a photoconductive TiOPc electrode. Liq. Cryst..

[14] Zou J., Yang Q., Hsiang E.-L., Ooishi H., Yang Z., Yoshidaya K., Wu S.T. (2021). Fast-response liquid crystal for spatial light modulator and LiDAR applications. Crystals.

[15] Residori S., Bortolozzo U., Huignard J.P. (2018). Liquid crystal light valves as optically addressed liquid crystal spatial light modulators: Optical wave mixing and sensing applications. Liq. Cryst. Rev..

[16] Manda R., Pagidi S., Lim Y.J., He R., Song S.M., Lee J.H., Lee G.-D., Lee S.H. (2019). Self-supported liquid crystal film for flexible display and photonic applications. J. Mol. Liq..

[17] Rudquist P. (2013). Orthoconic antiferroelectric liquid crystals. Liq. Cryst..

[18] Dąbrowski R., Kula P., Raszewski Z., Piecek W., Otón J.M., Spadło A. (2010). New orthoconic antiferroelectrics useful for applications. Ferroelectrics.

[19] D’havé K., Dahlgren A., Rudquist P., Lagerwall J.P.F., Andersson G., Matuszczyk M., Lagerwall S.T., Dąbrowski R., Drzewiński W. (2000). Antiferroelectric Liquid Crystals with 45◦ Tilt—A New Class of Promising Electro-Optic Materials. Ferroelectrics.

[20] Rudquist P., Meier J.G., Lagerwall J.P.F., D’have K., Lagerwall S.T. (2002). Tilt plane orientation in antiferroelectric liquid crystal cells and the origin of the pretransitional effect. Phys. Rev. E.

[21] Dąbrowski R., Gąsowska J., Otón J.M., Piecek W., Przedmojski J., Tykarska M. (2004). High tilted antiferroelectric liquid crystalline materials. Displays.

[22] Castillo P.L., Otón J.M., Dąbrowski R., Lara A., Quintana X., Bennis N. (2004). Electrooptics of antiferroelectric orthoconic reflective displays. Proc. SPIE.

[23] Drzewiński W., Czupryński K., Dąbrowski R., Neubert M. (1999). New antiferroelectric compounds containing partially fluorinated terminal chains. Synthesis and mesomorphic properties. Mol. Cryst. Liq. Cryst..

[24] Deptuch A., Górska N., Baran S., Urbańska M. (2024). Investigation of glass transition and polymorphism of smectogenic, partially fluorinated chiral compound by X-ray diffraction and infra-red spectroscopy. J. Mol. Liq..

[25] Żurowska M., Dąbrowski R., Dziaduszek J., Garbat K., Filipowicz M., Tykarska M., Rejmer W., Czupryński K., Spadło A., Bennis N. (2011). Influence of alkoxy chain length and fluorosubstitution on mesogenic and spectral properties of high tilted antiferroelectric esters. J. Mat. Chem..

[26] Żurowska M., Filipowicz M., Czerwiński M., Szala M. (2019). Synthesis and properties of ferro- and antiferroelectric esters with a chiral centre based on (S)-(+)-3-octanol. Liq. Cryst..

[27] Urbańska M., Morawiak P., Senderek M. (2021). Investigation of the tilt angle and spontaneous polarisation of antiferroelectric liquid crystals with a chiral centre based on (S)-(+)-3-octanol. J. Mol. Liq..

[28] Urbańska M., Perkowski P., Morawiak M., Senderek M. (2020). Antiferroelectric and ferroelectric mesophases created by (S) enantiomers with a short oligomethylene spacer and their usefulness in the formulation of orthoconic mixtures. J. Mol. Liq..

[29] Urbańska M., Strzeżysz O., Perkowski P. (2023). Fluorinated esters with a very broad temperature range of the antiferroelectric phase. Liq. Cryst..

[30] Levelut A.M., Germain C., Keller P., Liebert L., Billard J. (1983). Two new mesophases in a chiral compound. J. Phys..

[31] Furukawa K., Terashima T., Ichihashi M., Saitoh S., Miyazawa K., Inukai T. (1988). Chiral smectic C liquid crystals having an electronegative substituent ortho to the chiral tail group—A study of a factor determining the magnitude of spontaneous polarization. Ferroelectrics.

[32] Miyasato K., Abe S., Takezoe H., Fukuda A., Kuze E. (1983). Direct method with triangular waves for measuring spontaneous polarization in ferroelectric liquid crystals. Jpn. J. Appl. Phys..

[33] Schröder I.Z. (1893). Über die Abhängigkeit der Löslichkeit eines festen Körpers von seiner Schmelztemperatur. Z. für Phys. Chem..

[34] Van Laar J.J. (1908). Die Schmelz- oder Erstarrungskurven bei binären Systemen, wenn die feste Phase ein Gemisch (amorphe feste Lösung oder Mischkristalle) der beiden Komponenten ist. Z. für Phys. Chem..

[35] Chatelier H.L. (1884). On a general statement of the laws of chemical equilibrium. C. R. Acad. Sci..

[36] Cox R.J., Johnson J.F. (1978). Phase equilibria in liquid crystal mixtures. IBM J. Res. Dev..

[37] Bubnov A., Podoliak N., Hamplová V., Tomášková P., Havlíček J., Kašpar M. (2016). Eutectic behaviour of binary mixtures composed of two isomeric lactic acid derivatives. Ferroelectr..

[38] Żurowska M., Czerwiński M., Dziaduszek J., Filipowicz M. (2018). Examination of new chiral smectics with four aromatic rings. Phase Trans..

[39] Bubnov A., Hamplová V., Kašpar M., Vajda A., Stojanović M., Obadović D., Éber N., Fodor-Csorba K. (2007). Thermal analysis of binary liquid crystalline mixtures: System of bent core and calamitic molecules. J. Therm. Anal. Calori..

[40] Fitas J., Marzec M., Szymkowiak M., Jaworska-Gołąb T., Deptuch A., Tykarska M., Kurp K., Żurowska M., Bubnov A. (2018). Mesomorphic, electro-optic and structural properties of binary liquid crystalline mixtures with ferroelectric and antiferroelectric liquid crystalline behaviour. Phase Trans..

[41] Debnath A., Mandal P.K. (2017). Effect of fluorination on the phase sequence, dielectric and electro-optical properties of ferroelectric and antiferroelectric mixtures. Liq. Cryst..

[42] Lagerwall J.P.F., Saipa A., Giesselmann F., Dąbrowski R. (2004). On the origin of high optical director tilt in a partially fluorinated orthoconic antiferroelectric liquid crystal mixture. Liq. Cryst..

[43] Milewska K., Drzewiński W., Czerwiński M., Dąbrowski R., Piecek W. (2016). Highly tilted liquid crystalline materials possessing a direct phase transition from antiferroelectric to isotropic phase. Mater. Chem. Phys..

[44] Lalik S., Deptuch A., Fryń P., Jaworska-Gołąb T., Węgłowska D., Marzec M. (2019). Physical properties of new binary ferroelectric mixture. J. Mol. Liq..

[45] Chakraborty S., Das M.K., Bubnov A., Weissflog W., We D., Dabrowski R. (2019). Induced frustrated twist grain boundary liquid crystalline phases in binary mixtures of achiral hockey stick-shaped and chiral rod-like materials. J. Mater. Chem. C..

[46] Tykarska M., Kurp K., Zieja P., Herman J., Stulov S., Bubnov A. (2022). New quaterphenyls laterally substituted by methyl group and their influence on the self-assembling behaviour of ferroelectric bicomponent mixtures. Liq. Cryst..

[47] Żurowska M., Czerwiński M. (2017). The new high tilt mixtures with antiferroelectric phase at a broad temperature range and a long helical pitch. Liq. Cryst..

[48] Quintana X., Castillo P.L., Otón J.M., Bennis N., Lara A., Urruchi V., Dąbrowski R. (2004). Video-rate Multiplexed Driving Scheme for Passive Antiferroelectric Liquid Crystal Displays. Opto-Electr. Rev..

[49] Czerwiński M., de Blas M.G., Bennis N., Herman J., Dmochowska E., Otón J.M. (2021). Polymer stabilized highly tilted antiferroelectric liquid crystals—The influence of monomer structure and phase sequence of base mixtures. J. Mol. Liq..

[50] Takezoe H., Kondo K., Fukuda A., Kuze E. (1982). Determination of helical pitch in homeotropic cell of chiral Smectic C liquid crystal using center laser. Jpn. J. Appl. Phys..

[51] Kurp K., Tykarska M., Drzewicz A., Lapanik V., Sasnouski G. (2017). Effect of ferroelectric liquid crystalline quaterphenyl structure and handednesson helical pitch length in bicomponent mixtures. Liq. Cryst..

[52] Raszewski Z., Kędzierski J., Perkowski P., Piecek W., Rutkowska J., Kłosowicz S., Zieliński J. (2002). Refractive indices of the MHPB(H)PBC and MHPB(F)PBC antiferroelectric liquid crystals. Ferroelectrics.

[53] Furue H., Umeno E., Hatano J. (2005). Temperature Dependence of Helical Structure of Polymer-Stabilized Antiferroelectric Liquid Crystals. Mol. Cryst. Liq. Cryst..

[54] Chen J.H., Hsiue G.-H., Hwang C.-P., Wu J.-L. (1995). Broad liquid crystalline temperature range of ferroelectric liquid crystals. Liq. Cryst..

[55] Kelly S.M. (1989). Ferroelectric liquid crystals. Part 9. Laterally substituted phenyl benzoates incorporating a trans-1,4-disubstituted cyclohexane ring. Helv. Chim. Acta..

[56] Podoliak N., Hamplová V., Kašpar M., Novotná V., Glogarová M., Pociecha D., Górecka E. (2013). Highly tilted smectogens with bromine-substituted molecular core. Liq. Cryst..

[57] Fergusson K.M., Hird M. (2010). The dramatic influence of the location of bend and of lateral fluoro substitution on the mesomorphic properties of angular chiral esters based on a 1,3-disubstituted benzene ring. J. Mater. Chem..

[58] Gisse P., Cluzeau P., Ravaine V., Nguyen H.T. (2002). Characterization of a new chiral antiferroelectric liquid crystal with a lateral bromo substituent. Liq. Cryst..

[59] Novotná V., Hamplová V., Kašpar M., Glogarová M. (2002). New chlorine-substituted ferroelectric liquid crystals with four aromatic rings in the mesogenic core. Liq. Cryst..

[60] Yeap G.-Y., Hng T.-C., Yeap S.-Y., Górecka E., Ito M.M., Ueno K., Okamoto M., Mahmood W.A.K., Imrie C.T. (2009). Why do non-symmetric dimers intercalate? The synthesis and characterisation of the α-(4-benzylidene-substituted-aniline-4′-oxy)-ω-(2-methylbutyl-4′-(4″-phenyl)benzoateoxy)alkanes. Liq. Cryst..

[61] Podoliak N., Novotná V., Kašpar M., Hamplová V., Pacherová O. (2015). Chiral smectogens with four-phenyl-ring molecular core, laterally substituted by iodine atom. Liq. Cryst..

