# Supplementary material for: Synthesis and Characterization of New Chiral Smectic Four-Ring Esters
Source: Molecules. 2024 Jul 1;29(13):3134. doi: 10.3390/molecules29133134 (PMC11243116; doi:10.3390/molecules29133134)
Supplement: Supplementary file 1 [file molecules-29-03134-s001.zip › molecules-3049815-supplementary.pdf]

Supplementary materials for the article

Synthesis and Characterization of New Chiral Smectic Four-Ring Esters

MASS SPECTRA OF CHIRAL FOUR-RING ESTERS

The purity of the liquid crystalline esters was recorded using a Shimadzu prominence chromatograph. The strong molecular ion without hydrogen atom [M - H]$^-$ was observed; see Figures S1 and S2.

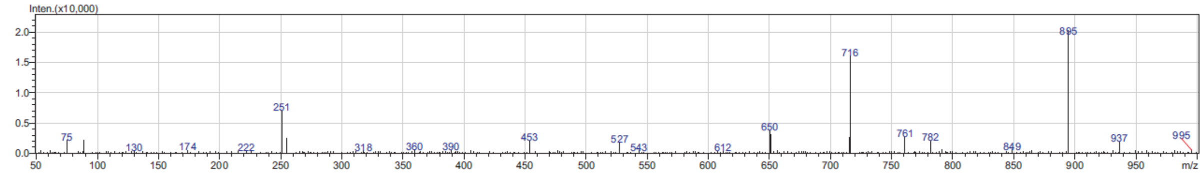

Figure S1. Mass spectrum of the compound **3PhPh**.

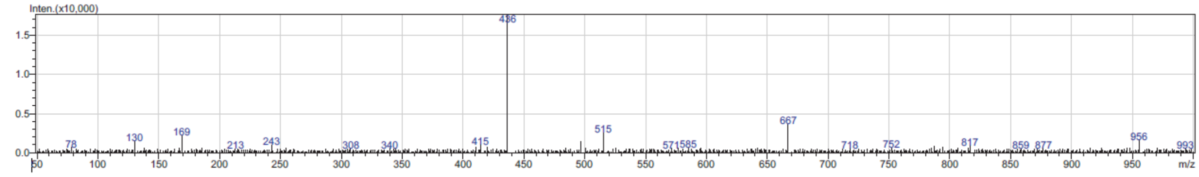

Figure S2. Mass spectrum of the compound **7PhPh**.

STRUCTURE CONFIRMATION OF CHIRAL FOUR-RING ESTERS

A comparison of the NMR spectra confirmed the compliance of real structures with the planned structures; see Figures S3-S6.

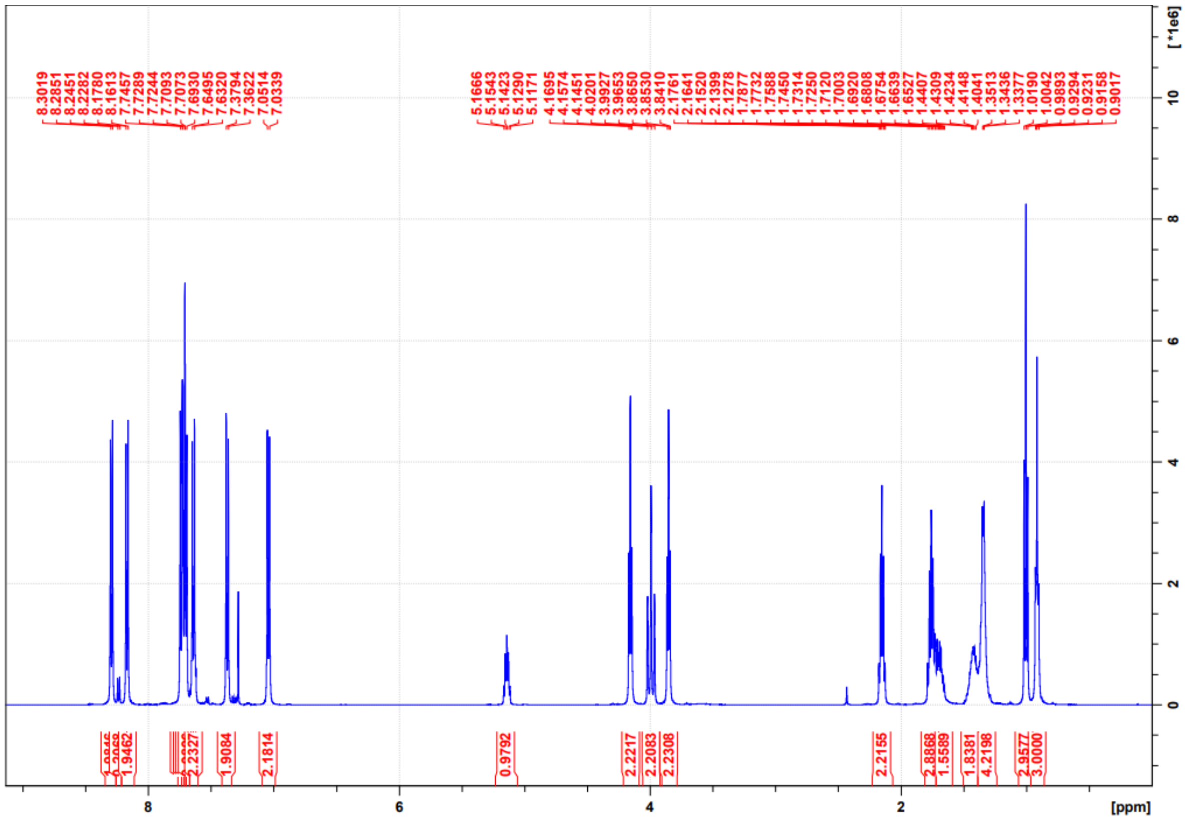

Figure S3. ^{1}H NMR spectrum of the compound **3PhPh** in $CDCl_3$.

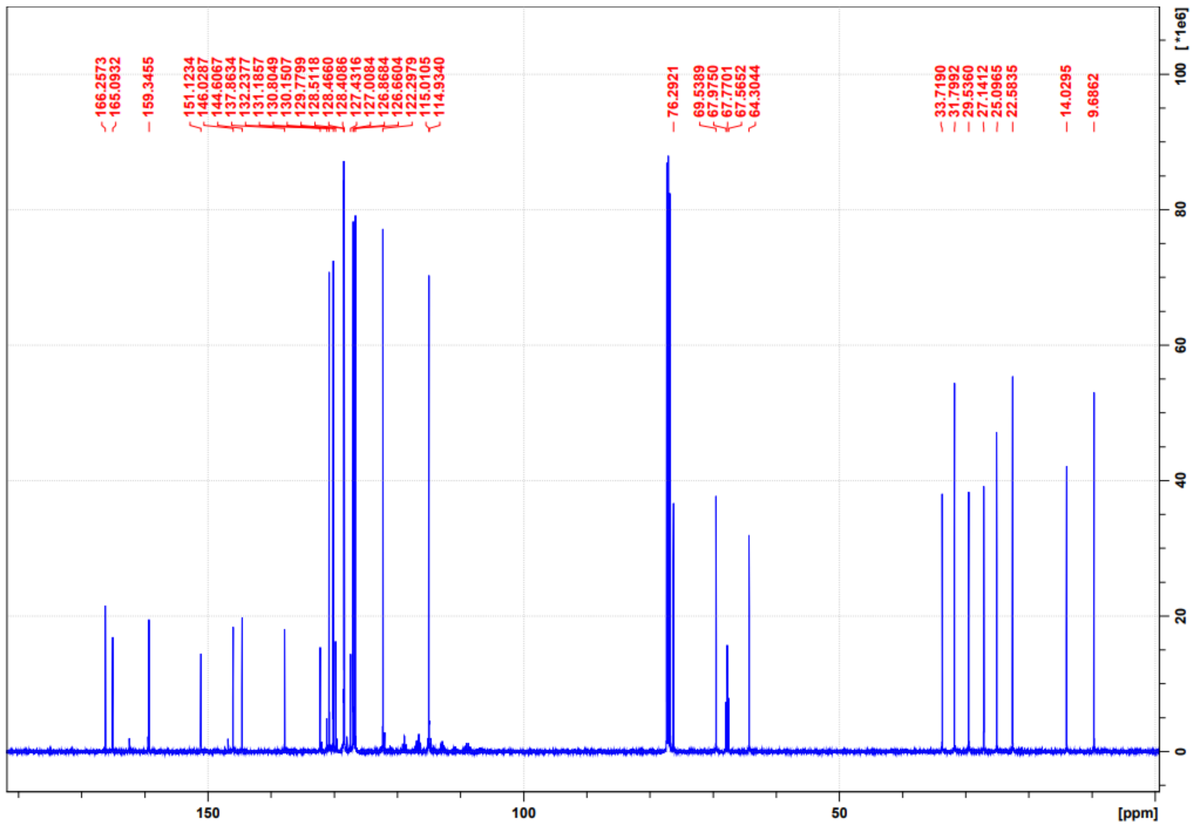

Figure S4. ^{13}C NMR spectrum of the compound **3PhPh** in $CDCl_3$.

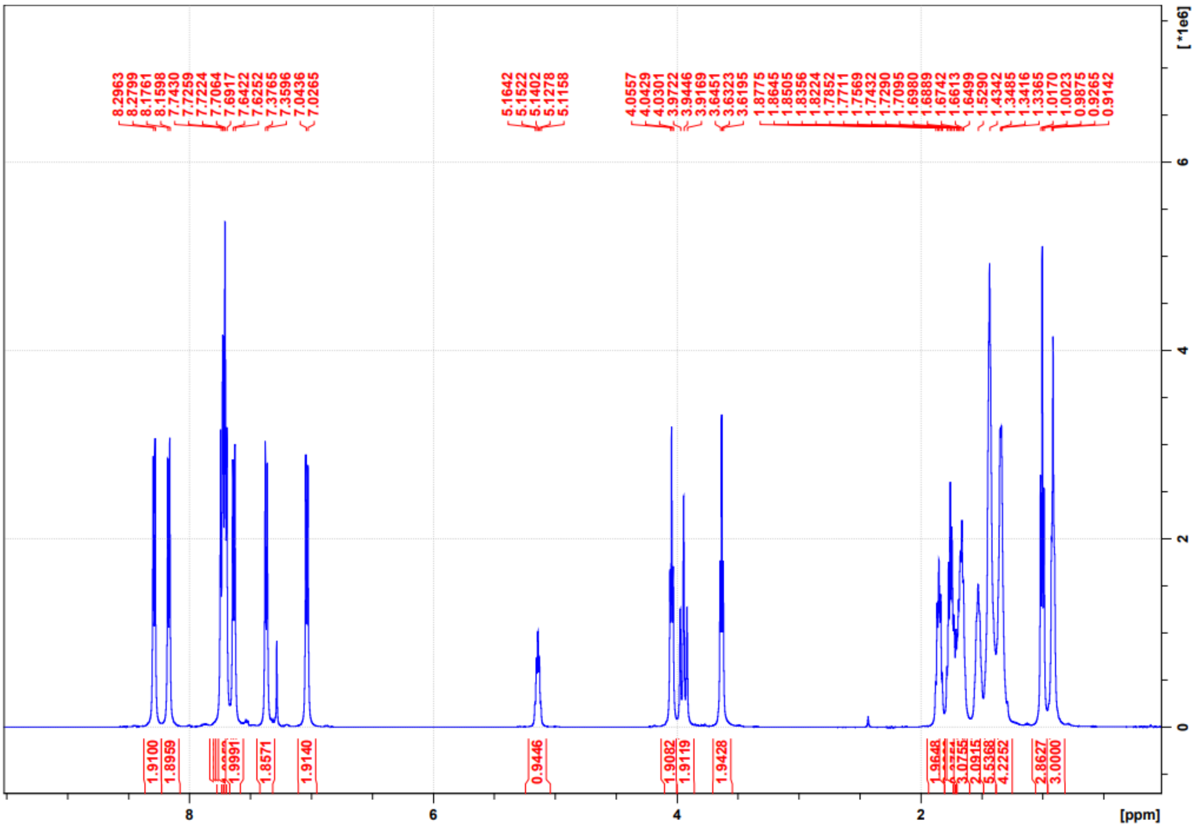

Figure S5. ^{1}H NMR spectrum of the compound **7PhPh** in $CDCl_3$.

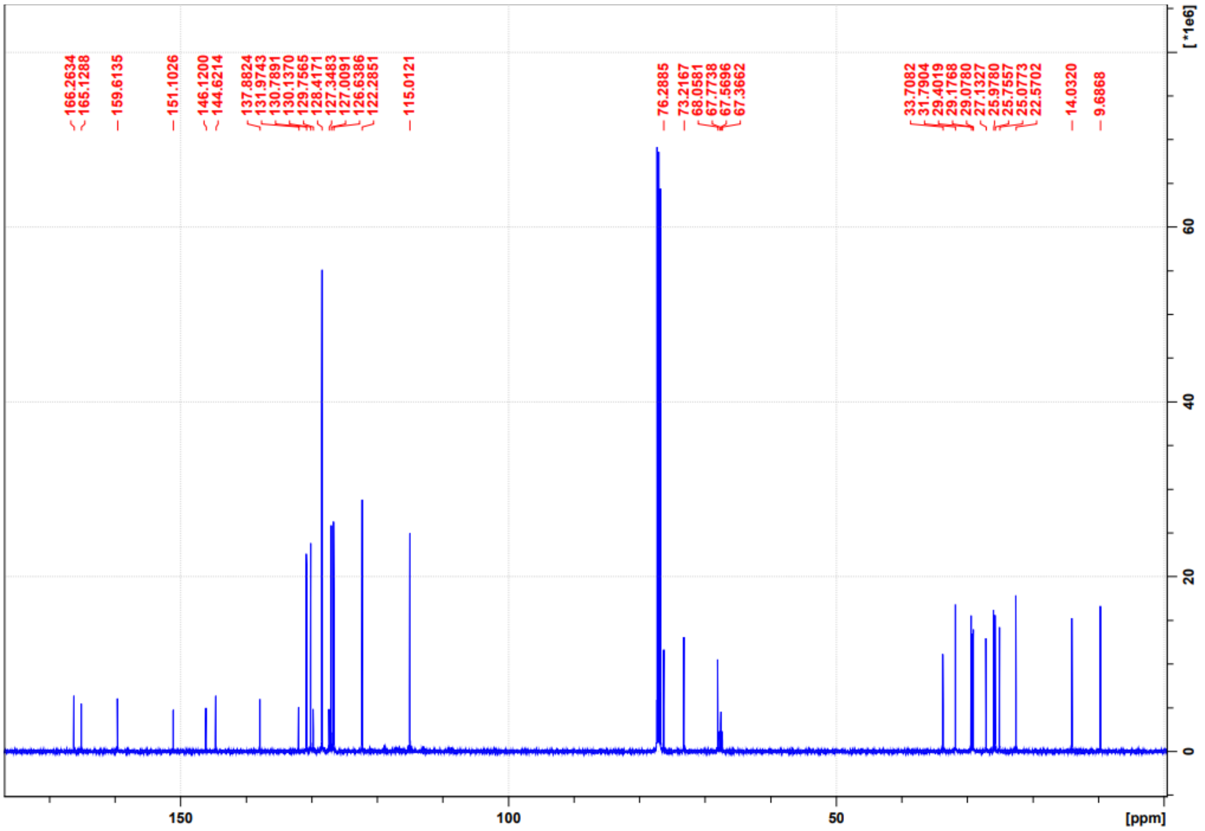

Figure S6. ^{13}C NMR spectrum of the compound **7PhPh** in $CDCl_3$.

DOUBLE HYSTERESIS CURVE OF DOPED MIXTURE

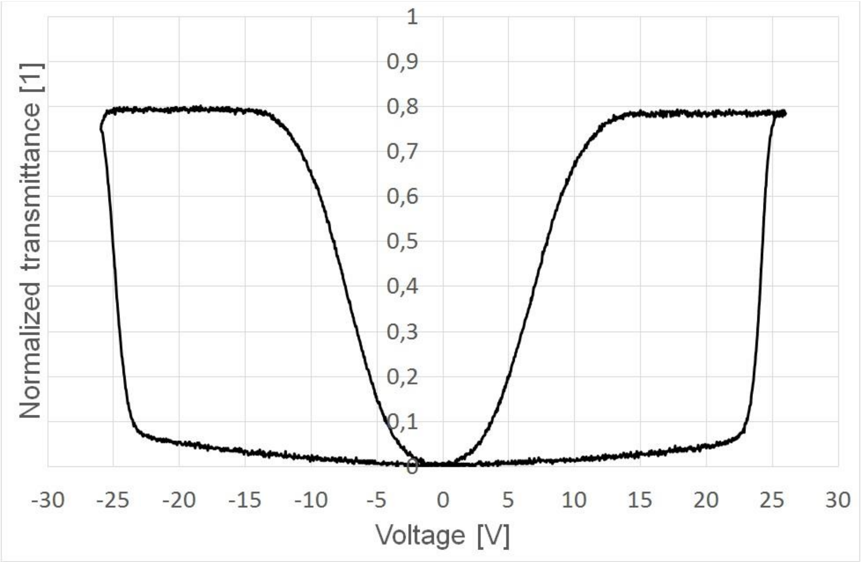

Figure S7. Quasistatic electro-optical response for the mixture W-450A, under application of a triangular waveform at 0.1 Hz.
